# Glutamine 89 is a key residue in the allosteric modulation of human serine racemase activity by ATP

**DOI:** 10.1038/s41598-018-27227-1

**Published:** 2018-06-13

**Authors:** Andrea V. Canosa, Serena Faggiano, Marialaura Marchetti, Stefano Armao, Stefano Bettati, Stefano Bruno, Riccardo Percudani, Barbara Campanini, Andrea Mozzarelli

**Affiliations:** 1 Dipartimento di Scienze degli Alimenti e del Farmaco, Università di Parma, Parma, Italy; 2 Istituto di Biofisica, Consiglio Nazionale delle Ricerche, Pisa, Italy; 3 Centro Interdipartimentale Biopharmanet-tec, Università degli Studi di Parma, Parma, Italy; 4 Dipartimento di Medicina e Chirurgia, Università di Parma, Parma, Italy; 5 Dipartimento di Scienze Chimiche, della Vita e della Sostenibilità Ambientale, Università di Parma, Parma, Italy

## Abstract

Serine racemase (SR) catalyses two reactions: the reversible racemisation of L-serine and the irreversible dehydration of L- and D-serine to pyruvate and ammonia. SRs are evolutionarily related to serine dehydratases (SDH) and degradative threonine deaminases (TdcB). Most SRs and TdcBs – but not SDHs – are regulated by nucleotides. SR binds ATP cooperatively and the nucleotide allosterically stimulates the serine dehydratase activity of the enzyme. A H-bond network comprising five residues (T52, N86, Q89, E283 and N316) and water molecules connects the active site with the ATP-binding site. Conservation analysis points to Q89 as a key residue for the allosteric communication, since its mutation to either Met or Ala is linked to the loss of control of activity by nucleotides. We verified this hypothesis by introducing the Q89M and Q89A point mutations in the human SR sequence. The allosteric communication between the active site and the allosteric site in both mutants is almost completely abolished. Indeed, the stimulation of the dehydratase activity by ATP is severely diminished and the binding of the nucleotide is no more cooperative. Ancestral state reconstruction suggests that the allosteric control by nucleotides established early in SR evolution and has been maintained in most eukaryotic lineages.

## Introduction

**Figure 1:**
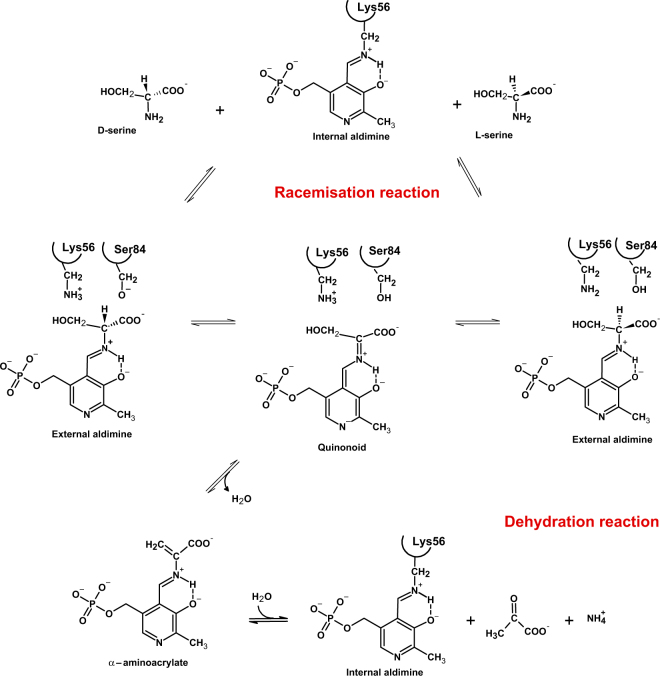
Reaction mechanism of SR. The incoming amino acid, either D- or L-Ser, in the unprotonated form, attacks the internal aldimine formed by PLP and Lys56 to generate an external aldimine. Lys56 in the case of L-Ser and Ser84 in the case of D-Ser extract the α-proton forming a carbanion intermediate that might tautomerize to quinonoid, although a deprotonated pyridine nitrogen makes PLP an inefficient electron sink. Reprotonation on the opposite face of the carbanion leads to racemisation. Alternatively, the β-elimination of a water molecule (a reaction commonly referred to as dehydration) leads to the formation of α-aminoacrylate that promptly decomposes to pyruvate and ammonia. Neither quinonoid nor α-aminoacrylate intermediates have ever been experimentally observed.

Serine racemase (SR) (EC 5.1.1.18) is the enzyme responsible for the synthesis of D-serine, the natural co-agonist of N-methyl-D-aspartate (NMDA) receptors^1^. It has been identified and characterized in mammals^2–7^, salamander^8^, plants^9–13^, fission yeast^14,15^, and amoebae^16,17^. Only 9 out of the 88 EC 5.1.1.18 UniProtKB entries have been manually annotated and reviewed, indicating that most of them are predicted SRs. SR is a dimeric pyridoxal 5′-phosphate (PLP)-dependent enzyme and, like many PLP-dependent enzymes, it is able to catalyse secondary reactions on its natural substrate, the most relevant of which is the β-elimination, or dehydration, of both L- and D-serine^3,5^ (Fig. 1). The catalytic efficiency (k_cat_/K_m_) for L-serine racemisation *in vitro* is extremely low, about 9 s^−1^∙M^−1^ for the human enzyme hSR (see below and^18^). For comparison, bacterial alanine racemase is about 50-fold more efficient at catalysing the racemisation of L-alanine^19^. However, SR activity *in vivo* is able to efficiently sustain D-serine production, since SR knockout mice exhibit D-serine levels in brain that are less than 10% of normal amounts^20,21^. hSR is equally efficient in the catalysis of L-serine dehydration (k_cat_/K_m_ = 8 s^−1^∙M^−1^). The efficiency of this reaction is increased by about 30-fold by ATP, whereas the efficiency for L-serine racemisation is increased only 2-fold (see below and^18^). Under physiological conditions, SR is believed to be fully saturated by ATP and the reason for this unbalance towards the dehydration reaction is still unknown. It was suggested that both activities contribute to D-serine homeostasis, particularly in the brain areas lacking the main degradative enzyme for D-amino acids, D-amino acid oxidase (DAAO)^5^. Moreover, modulation of racemisation activity by cellular localization, post-translational modifications or interaction with other proteins are likely to occur. It is well assessed that eukaryotic serine racemases and serine dehydratases (SDH) share a common heritage^4^, suggesting that the racemase activity might have arisen as a secondary reaction, leading to the ability of the cell to produce D-serine.

The allosteric control of SR activity by ATP has been documented in the human (hSR)^3,18^, mouse, rat (*Rn*SR) and yeast (*Sp*SR)^14^ enzyme, whereas data on the plant enzyme are contradictory. *Arabidopsis thaliana* and *Hordeum vulgare* enzymes are not activated by ATP^9,10,22^ and a single report on *Oryza sativa* SR indicates a slight, possibly negligible, inactivation by ATP^12^. However, plant SRs represent a distinct group in eukaryotic serine racemases^9^ and are thus likely to be all insensitive to nucleotide binding. Interestingly, the communication between active site and the ATP binding site is bidirectional, with the affinity for ATP increasing in the presence of active site ligands such as glycine and malonate^18,23^. The occupation of the active site by substrates and/or inhibitors leads to conformational changes in the small domain, which rotates with respect to the large domain to restrain accessibility to the active site^14,24^. This domain movement, i.e. the transition from an open to a closed structure, is crucial for the racemisation activity. Indeed, Ser84, which is essential for L-Ser isomerisation and D-Ser dehydration, belongs to the small domain, and is not involved in L-Ser dehydration (Fig. 1)^25^. The domain movement upon substrate binding is essential to position this residue correctly for Cα protonation to occur^24^. The allosteric control by ATP on SR activity is likely exerted through both tertiary and quaternary effects. As a matter of fact, ATP binding to *Sp*SR causes changes in the relative orientation of the subunits^14^ and the binding of the nucleotide to the human enzyme is cooperative, with a Hill coefficient of about 2^18^, the maximum value predicted for a protein with two binding sites. On the other hand, the observed differences in the relative modulation of racemase and dehydratase activities by ATP are likely due to tertiary conformational changes transmitted from the allosteric site to the active site. The presence of a H-bond network connecting PLP to ATP was originally suggested by Goto and coworkers, who co-crystallized *Sp*SR as a complex with a stable analogue of ATP, adenosine 5′-(β,γ-methylene]) triphosphate (AMP-PCP)^14^. The network connects the O3′ of PLP with the ribose hydroxyl groups and γ-phosphate of AMP-PCP and comprises M53, N84, Q87, E281 and N311 (which correspond to T52, N86, Q89, E283 and N316 in the sequence of hSR). We observed that these residues, with the exception of M53, are also present in the close structural homolog of SR bacterial degradative threonine deaminase (TdcB), whose activity is also regulated by nucleotides. In this work, phylogenetic analyses and functional characterization allowed to identify Q89 as a key residue in the allosteric communication between the active and allosteric sites of hSR.

## Results

### Identification of key residues for the allosteric communication between active site and ATP-binding site

**Figure 2:**
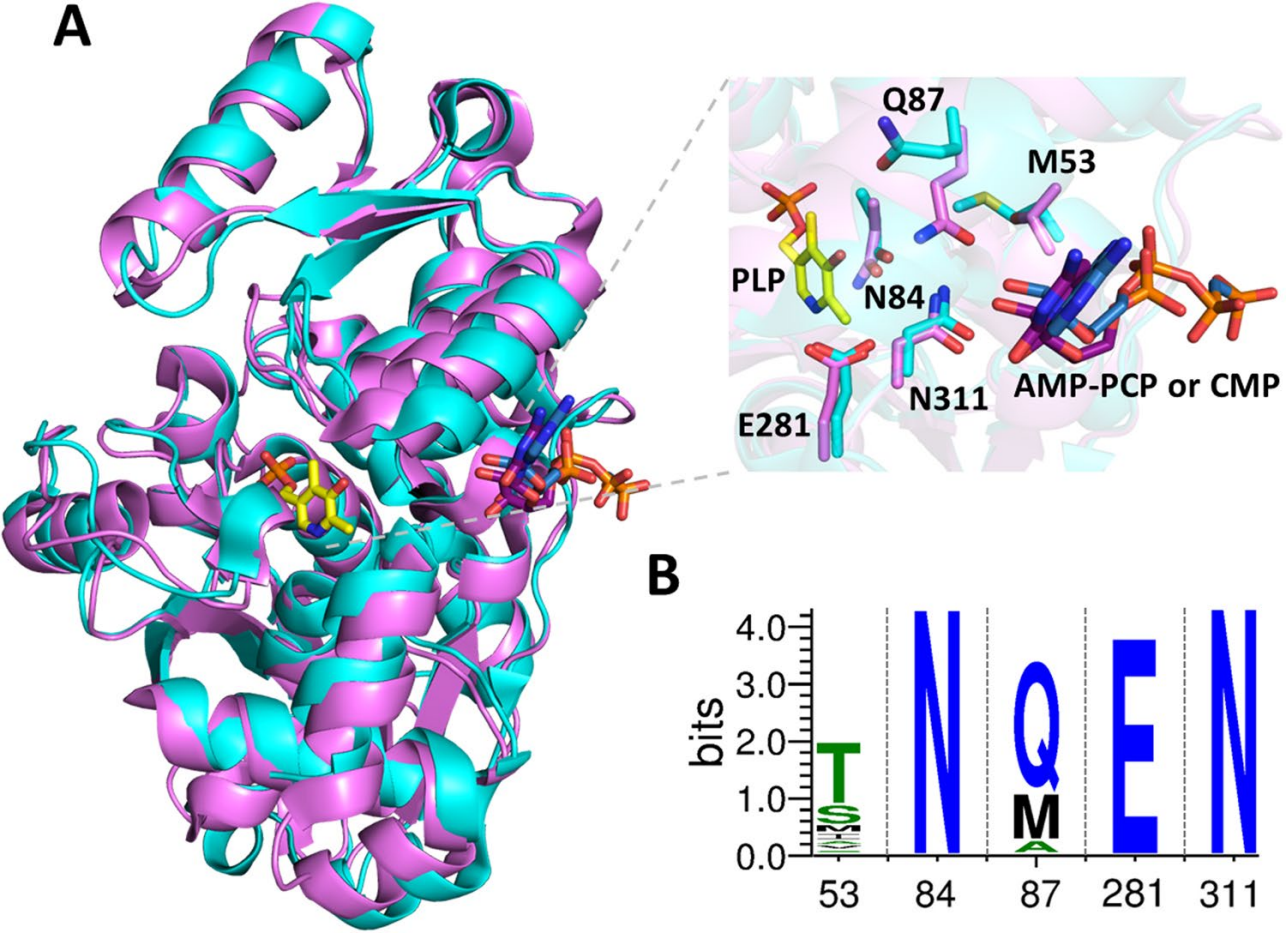
Identification of residues involved in the formation of a H-bond network connecting the active site and the ATP binding site in SR. (**A**) Structural alignment of SR with bacterial threonine deaminase. The three-dimensional structure of *Sp*SR in complex with AMP-PCP (cyan; pdb code: 1WTC) was superimposed to the three-dimensional structure of *St*TdcB in complex with CMP (pink; pdb code: 2GN2) using the built-in tool of Pymol. Inset reports a close up of the nucleotide binding site with PLP, nucleotide and residues forming the H-bond network shown in stick mode. Numbering is based on *Sp*SR sequence. *Sp*SR Q87 corresponds to Q89 in hSR sequence. (**B**) Logo plot of the alignment of the five amino acid residues of the H-bond network showing the reduction of uncertainty due to sequence conservation. The sequences analysed were from SR, SDH and TdcB of several organisms as detailed in Figure S1.

The closest structural homologs of *Sp*SR and hSR are the degradative threonine dehydratase from *Salmonella enterica* subsp. *enterica serovar* Typhimiurium (*St*TdcB)^26^ (pdb code: 2GN2) and human serine dehydratase (hSDH)^27,28^ (pdb code: 1P5J)^14^. SR, TdcB and SDH are all able to catalyse the cleavage of the Cβ-O bond of either serine or threonine with formation of an α-aminoacrylate/α-aminocrotonate that spontaneously hydrolyses to pyruvate/α-ketobutyrate and ammonia (Fig. 1). *St*TdcB catalyses the first step of L-Thr degradation to propionate^29^ and is activated by AMP and CMP, with a 7.7- fold decrease in K_m_ and a 3-fold increase in V_max_ in the presence of 5 mM AMP. Nucleosides monophosphate are also responsible for the stabilisation of a tetrameric structure of the enzyme^26^. On the other hand, the control of hSDH activity by nucleotides has never been reported and an ATP-binding site is not detectable in human SDH^30^. The superposition of the structure of *Sp*SR in complex with AMP-PCP with the structure of *St*TdcB in complex with CMP shows a very good overlapping, with an RMSD of 2.54 Å and an excellent matching of the bound nucleotide (Fig. 2A). In addition, the residues that form the hydrogen bonding network linking the O3′ of PLP to the ribose hydroxyl groups of the nucleotide, namely M53, N84, Q87, E281 and N311 in the *Sp*SR structure (pdb code: 1WTC)^14^, are identical in the two proteins, with the exception of M53 that is substituted by a Thr residue in *St*TdcB (Fig. 2A). To assess the conservation of these residues in SR structural and functional analogues, we aligned 184 sequences of SR, SDH and TdcB from different species (see Fig. S1 for details on the sequences and organisms) and calculated the uncertainty (Shannon entropy) for each of the five residues forming the H-bond network in *Sp*SR. The uncertainty is zero for N84 and E281 and close to zero (0.1) for N311, indicating that these residues are strictly conserved (Fig. 2B). On the other hand, position 53 is highly variable and accommodates Thr, Met, Ile, Ala, and Val. Uncertainty at position 87 (89 in hSR numbering) is lower than that observed for position 53, and the observed residues (either Gln, Met, or Ala) show a clear pattern: in all the proteins whose activity is controlled by nucleotides (i.e. mammalian and yeast SR and TdcB), this position is occupied by Gln (see also Fig. S2). Met is observed for SDH and Ala is consistently observed in plant SRs. Q87 of *Sp*SR in the form bound to AMP-PCP not only participates in the H-bond network that connects the active site to the ATP-binding site, but also interacts through water molecules with the mobile arginine loop (Asn84) which recognizes the substrate carboxylate. The corresponding Ala and Met residues in plant SR and in SDH are unable to form a H-bond with their side chains and should thus interrupt the H-bond network. Based on these considerations, the Q89M and Q89A variants of hSR (Q89M-hSR and Q89A-hSR) were prepared and the effect of the mutations on the allosteric modulation of activity by ATP was assessed.

### Characterization of Q89M and Q89A variants

**Figure 3:**
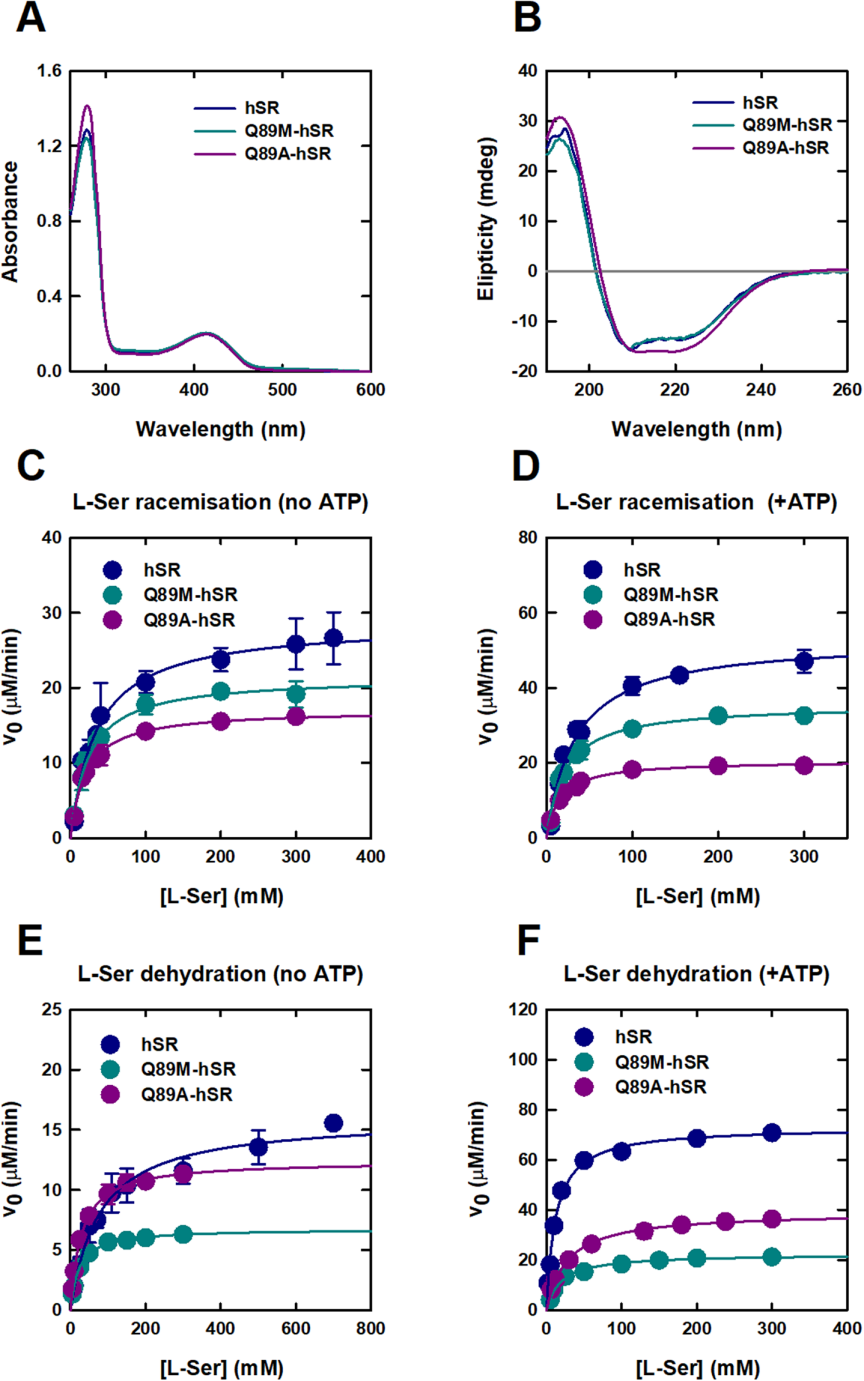
Effect of Q89 substitutions on hSR structure and catalytic parameters. Absorbance (**A**) and far-UV circular dichroism (**B**) spectra of wt hSR and Q89 mutants. The dependences of the initial rate for L-Ser racemisation (**C** and **D**) and dehydration (**E** and **F**) on L-Ser concentration were fitted to equation (3). Calculated kinetic parameters are reported in Table 1. Dependences were collected either in the presence (**D** and **F**) or absence (**C** and **E**) of 2 mM ATP. For comparison purposes, velocity data were normalized to 0.4 μM enzyme concentration. For some data error bars are not visible since they are smaller than the data points.

Q89M-hSR and Q89A-hSR gave an expression yields in *Escherichia coli* of about 4 mg and 1.3 mg for each liter of culture, respectively. In the case of Q89M-hSR the yield is only slightly lower than that observed for wild-type hSR (about 6 mg/l), on the contrary Q89A-hSR was significantly less stable and the protein precipitated easily upon concentration. The expression yields of wt hSR and Q89M-hSR are relatively high, if compared to those reported by us and other groups^5,31–33^ on similar constructs. The co-expression of His-tagged hSR with chaperones led to a more than 2-fold increase in the expression yields, comparable with the recently reported yields obtained by tagging hSR with the solubility tag maltose binding protein^33^. The purity of Q89M-hSR preparation was 94%, comparable to that obtained for wt hSR (98%) (Fig. S3). The purity of Q89A-hSR was 74% (Fig. S3). The different purity of the latter preparation was ascribed to the lower solubility of the mutant. However, since the concentration of the protein was estimated from the absorbance peak of the cofactor, it was possible to collect reliable activity data. The absorbance spectrum and the secondary structure of Q89M-hSR are identical to those of the wt protein, as estimated by UV-Vis and far-UV circular dichroism spectroscopy (Fig. 3A,B). For Q89A-hSR the absorbance spectrum has a relatively more intense peak at 280 nm due to the lower homogeneity of the preparation. However, the peak of the cofactor is correctly centred at 412 nm. The presence of contaminating proteins is also the likely origin of a slightly different CD spectrum. These findings indicate that the mutations do not significantly affect either the global folding or the local structure of the active site as demonstrated by the total retention of the catalytic efficiency of the proteins in the absence of ATP (Table 1).

**Table 1:** Catalytic parameters (±s.e.m.) for the dehydration of L-Ser and D-Ser and racemisation of L-Ser by hSR, Q89M-hSR and Q89A-hSR.

Reaction	Protein	K_m_ (mM)		k_cat_ (min^−1^)		k_cat_/K_m_ (s^−1^·M^−1^)		Increase
		−ATP	+ATP	−ATP	+ATP	−ATP	+ATP	
L-Ser racemisation	hSR	35 ± 4	34 ± 5	19 ± 1	35 ± 1	9.2 ± 1.0	17.5 ± 2.4	1.9
	Q89M-hSR	24 ± 2	22 ± 2	14 ± 1	24 ± 1	9.9 ± 0.8	18.0 ± 2.0	1.8
	Q89A-hSR	20 ± 2	16 ± 1	11 ± 1	14 ± 1	9.4 ± 0.8	14.7 ± 0.9	1.6
L-Ser dehydration	hSR	76 ± 10	12 ± 1	37 ± 4	183 ± 3	8.1 ± 1.3	253.0 ± 19.6	31
	Q89M-hSR	22 ± 1	19 ± 2	17 ± 1	56 ± 1	12.7 ± 0.6	48.9 ± 4.5	3.9
	Q89A-hSR	28 ± 2	27 ± 3	31 ± 1	98 ± 1	18.1 ± 0.6	59.4 ± 3.6	3.3
D-Ser dehydration	hSR	46 ± 3	167 ± 16	1.7 ± 0.1	23 ± 1	0.6 ± 0.1	2.4 ± 0.2	4.0
	Q89M-hSR	60 ± 2	65 ± 7	3.2 ± 0.1	9.6 ± 0.3	0.9 ± 0.1	2.5 ± 0.4	2.8
	Q89A-hSR	n.d.	n.d.	n.d.	n.d.	1.6 ± 0.2	3.6 ± 0.3	2.3

Due to the low purification yields of Q89A-hSR and the high concentration required for the D-Ser dehydration reaction only the k_cat_/K_m_ was estimated. The dependences of initial velocities on substrate concentration used to calculate the kinetic parameters are shown in Fig. 3. The last column reports fold increase of catalytic efficiency (k_cat_/K_m_) in the presence of ATP with respect to the absence of nucleotide.

### Activation of dehydration by ATP is dramatically reduced in Q89-variants in comparison to wild-type SR

**Figure 4:**
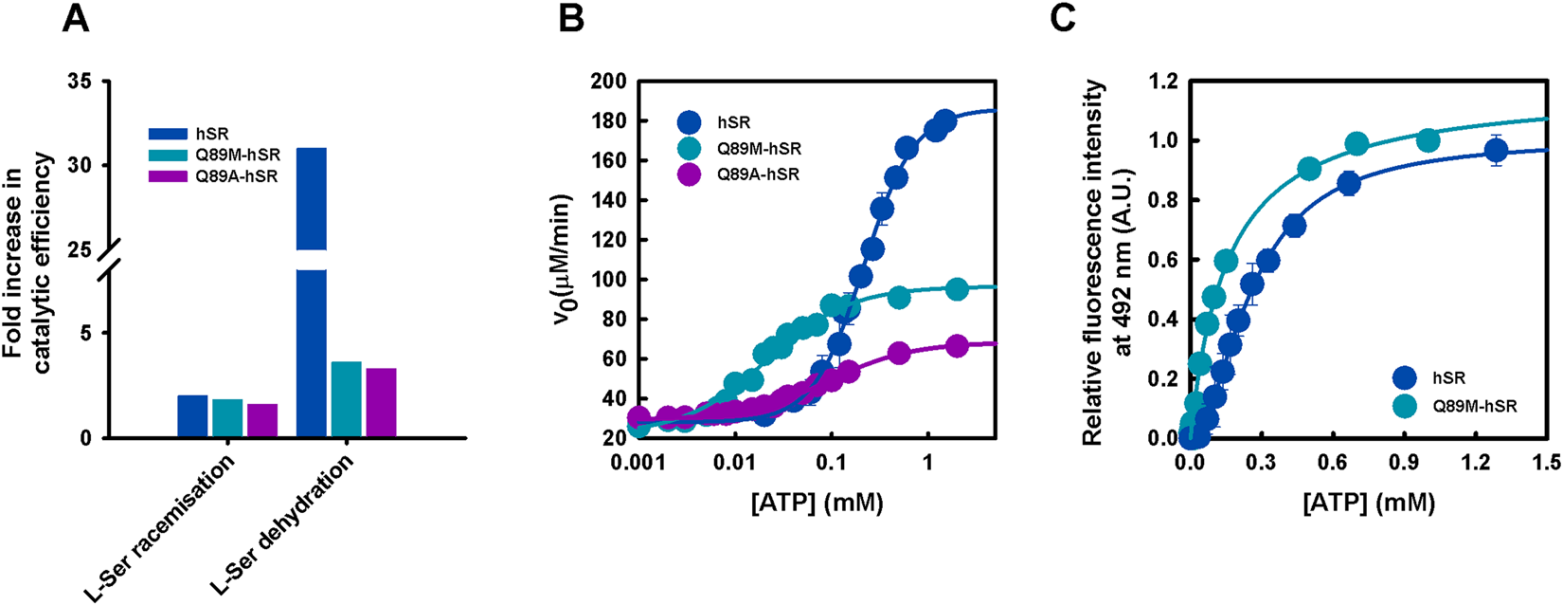
Effect of Q89 substitutions on ATP binding to SR and ATP-dependent modulation of activity. (**A**) Fold-increase in the catalytic efficiency of wt hSR and Q89 mutants for the racemisation and dehydration reactions. Activity was measured in the presence and absence of 2 mM ATP and 300 mM L-Ser for Q89M-SR and 500 mM L-Ser for wt hSR. (**B**) The half maximal effective concentration of ATP (EC_50_) for Q89M-hSR and Q89A-hSR was calculated by fitting the dependence of the L-Ser dehydratase activity on the concentration of ATP by equation (1). EC_50_ is 21 ± 2 μM and 91 ± 5 μM for Q89M-hSR and Q89A-hSR, respectively. The dependence for hSR (taken from^18^) was fitted to a sigmoidal equation with EC_50_ = 220 ± 10 μM, n = 1.7 ± 0.2. (**C**) Determination of the dissociation constant of ATP for Q89M-hSR by fluorimetric titrations. The dependence of the emission intensity of the cofactor on the concentration of ATP was fitted to equation (2) with a K_D_ of 154 ± 13 μM. The dependence for wt hSR (taken from^18^) was fitted to a sigmoidal equation with EC_50_ = 257 ± 10 μM, n = 1.8 ± 0.1.

Preliminary experiments showed that the specific dehydratase activity of the mutants increased only by about 4-fold by addition of 2 mM ATP, a saturating concentration for the wt enzyme (data not shown). Under the same conditions the activity of wt hSR increased 7-fold^18^. The activity of the mutants was not increased by further addition of ATP up to 10 mM concentration, suggesting that the lower activation observed for the mutants is not due to a decrease in the affinity for ATP. Since changes in ATP binding might affect the K_m_ for L-Ser, the dependence of the initial rate of dehydration on the concentration of L-Ser was determined in the presence and absence of 2 mM ATP. Similar data were acquired for L-serine racemisation (Fig. 3C–F) and D-serine dehydration (Fig. S4). Data fitting to the Michaelis-Menten equation yielded the parameters reported in Table 1. Due to the low yields of Q89A-hSR only the catalytic efficiency for the dehydration of D-serine was calculated both in the presence and absence of ATP (Table 1). Interestingly, the catalytic efficiency for all three reactions in the absence of ATP for the two mutant proteins is similar to that of the wt protein, demonstrating that the reduced influence on the dehydration reaction is not due to a nonspecific effect of the mutations on the catalytic integrity of the protein. The effect of ATP on Q89M-hSR and Q89A-hSR is significantly different with respect to wt hSR. K_m_ for all the measured activities is not affected by ATP and k_cat_ increases slightly (Table 1). As a result, catalytic efficiency only increases by up to 3.9-fold (Fig. 4A) for Q89M-hSR and Q89A-hSR. In contrast, a 31-fold increase in the catalytic efficiency of L-Ser dehydration was measured for wt hSR. Since ATP affects both K_m_ and k_cat_ for L-Ser dehydration of hSR, the consequence of nucleotide binding is more evident on the catalytic efficiency than on the specific activity.

### ATP binding to Q89M-hSR and Q89A-hSR is non-cooperative

Based on the catalytic parameters calculated for L-Ser dehydration we could set the experimental conditions to measure ATP binding to Q89M-hSR and Q89A-hSR by activity assays working at saturating L-Ser concentrations. Under these conditions, the increase in activity as a function of ATP concentration only depends on ATP binding. ATP binding, measured in the presence of saturating 300 mM L-Ser (Fig. 4B), revealed to be non-cooperative with a half-maximal activation (EC_50_) of 21 ± 2 μM for Q89M-hSR and 92 ± 6 μM for Q89A-hSR. These results are in marked contrast with those collected on wt hSR, where the ATP binding exhibited a lower affinity (EC_50_ = 220 ± 10 μM) and was highly cooperative (n = 1.7) (Fig. 4B). Overall the results collected on the two mutants are very similar, suggesting that, as predicted by the conservation analysis, both Met and Ala are equally able to interrupt the H-bond network that allows allosteric control by ATP. However, the Gln→Ala substitution seems to affect the overall stability of the protein to a larger extent than the Gln→Met, as indicated by the lower expression/purification yields and by a lower stability in the experimental buffer (data not shown). For these reasons we decided to perform subsequent analysis only on the Q89M mutant. ATP binding was directly determined on Q89M-hSR in the absence of substrate by monitoring the intensity of PLP fluorescence emission upon excitation at 412 nm^18^. ATP binding causes a 1.65-fold increase in fluorescence emission intensity. Fitting of the dependence of fluorescence intensity at 492 nm on ATP concentration gave a dissociation constant of 154 ± 13 μM, with no cooperativity (Fig. 4C and Table 2). The 6-fold difference between K_D_ and EC_50_ might be attributed to binding of ATP to different conformations of the protein preferentially stabilised in the absence and presence of substrate.

**Table 2:** Dissociation constants (±s.e.m.) for ligands of wt hSR and Q89M-hSR calculated by fluorimetric titrations.

Ligand 1	Ligand 2	K_D_ (μM)	
		hSR	Q89M-hSR
ATP	/	250 ± 4	154 ± 13
	Glycine	4.9 ± 0.6	48 ± 5
Glycine	/	7000 ± 300	110 ± 2
	ATP	470 ± 30	28 ± 3

Dissociation constants for hSR were taken from^18^.

### Q89M mutation interferes with the allosteric communication between the active site and the ATP binding site

**Figure 5:**
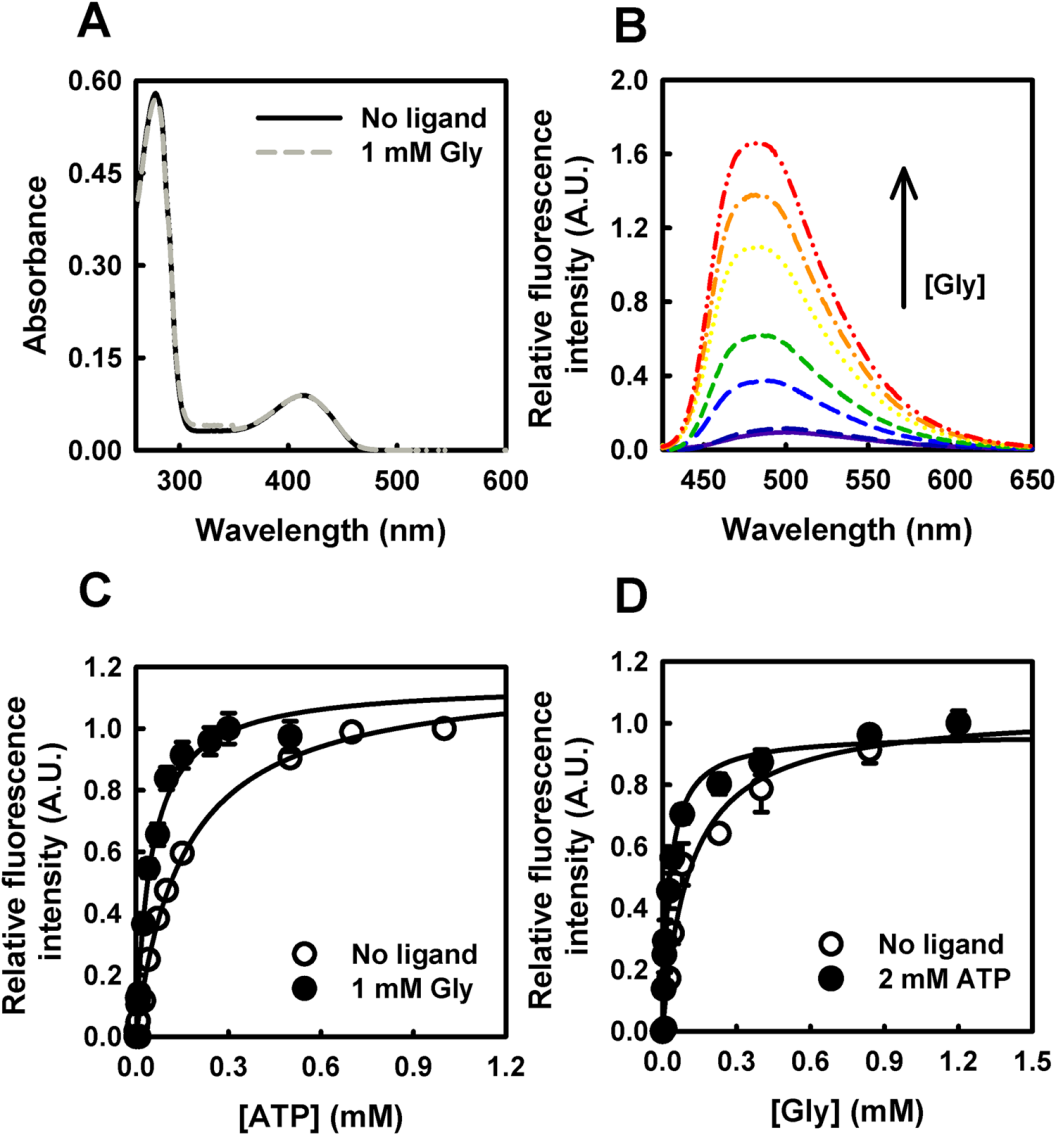
Effect of Q89M mutation on the allosteric communication probed by glycine binding. (**A**) Absorbance spectra of Q89M-hSR in the presence and absence of 1 mM Gly. (**B**) Fluorescence emission spectra of Q89M-SR in the absence of ATP and in the presence of increasing concentrations of Gly from 0.01 to 1.2 mM. (**C**) Dependence of the fluorescence emission intensity at the peak maximum on the concentration of ATP, in the absence and presence of 1 mM Gly. Fitting of the data to equation (2) gives a K_D_ for ATP of 154 ± 13 μM (same data as in Fig. 4C, Q89M-hSR) and 48 ± 5 μM in the absence and presence of glycine, respectively. (**D**) Dependence of the fluorescence emission intensity at the peak maximum on the concentration of glycine, in the absence and presence of 2 mM ATP. Fitting of the data to equation (2) gives a K_D_ for glycine of 110 ± 2 μM and 28 ± 3 μM in the absence and presence of ATP, respectively.

Glycine is a substrate analogue of SR, that is able to form an external aldimine with PLP which in turn is unable to proceed along the catalytic pathway. For this reason, glycine behaves as an unproductive substrate. In the case of hSR, binding of either ATP or glycine to their site strongly increases the affinity of the ligand at the other site^18^, indicating the existence of a crosstalk between the active site and the ATP-binding site. Furthermore, binding of ATP to glycine-saturated hSR is non-cooperative, an indication that glycine stabilises a conformation of the enzyme having a high affinity for ATP. As in the case of ATP, binding of glycine to the active site of hSR can also be followed by changes in the fluorescence emission of the cofactor^18^. Glycine binding to hSR with formation of an external aldimine was accompanied by very small changes in the absorbance spectrum (Fig. 5A), whereas it caused a significant increase in the fluorescence emission spectrum upon excitation at 412 nm (Fig. 5B). Titration with ATP of glycine-saturated Q89M-hSR led to a further increase in fluorescence emission, similar to the one registered on hSR in the absence of glycine. The K_D_ of ATP for Q89M-hSR saturated with 1 mM glycine is 48 ± 5 μM (Fig. 5C and Table 2), comparable to the EC_50_ measured by activity assays. This finding further strengthens the observation that the affinity of ATP is influenced by the presence of a ligand at the active site. The K_D_ for glycine decreases from 110 ± 2 μM to 28 ± 3 μM when ATP is bound to the enzyme (Fig. 5D and Table 2). It is remarkable the large increase in glycine affinity of Q89M-hSR with respect to wt hSR, both in the absence and presence of ATP (Table 2). This finding suggests that Q89M-hSR active site might be in a closed conformation. To summarize, the Q89M mutation has two effects on ligand binding: it affects the intrinsic affinity and prevents the allosteric communication. The effect on the intrinsic affinity is similar for glycine and ATP: in the absence of any other ligand, both glycine and ATP exhibit a higher affinity for Q89M-hSR with respect to wt hSR. On the other hand, the mutation has reduced the bidirectional allosteric communication between the active site and the ATP-binding site, as demonstrated by the almost negligible stimulatory effect of the binding of one ligand on the binding of the other one.

## Discussion

Catalytic promiscuity is a general property of many enzymes and is linked to their ability to evolve new reaction specificities^34^. PLP-dependent enzymes are renowned for their ability to catalyse secondary reactions in addition to the main one, as is the case for the β-elimination catalysed by transaminases. In hSR this aspect is particularly relevant, since the reaction that is considered the secondary one, i.e. L-Ser dehydration, is as efficient as the main one. Therefore, understanding the molecular and evolutionary basis of the duplex catalytic activity is of great relevance to the understanding of the physiology of the enzyme. This, in turn, might be exploited in the development of ligands that modulate hSR activity for a therapeutic action^35,36^. One interesting aspect of the dehydratase activity of hSR is that it is very specifically stimulated by ATP, and, to a lesser extent, by CTP and GTP, in such a way that when the enzyme is saturated with ATP, i.e. under physiological conditions, the dehydratase activity is 15-fold more efficient than the racemisation (Table 1). Furthermore, since ATP binding to hSR is highly cooperative, the modulation of enzyme activity by the nucleotide involves both a quaternary rearrangement favouring a tetrameric structure^37^, and a tertiary change of the active site conformation that causes an increase in the efficiency of L-Ser dehydration over racemisation.

Here, we have demonstrated that glutamine 89 plays a critical role in the modulation of hSR activity by nucleotides. Interestingly, Q89M and Q89A mutations equally affect both the cooperativity of ATP binding and the control that ATP exerts on dehydratase activity. As expected based on evolutionary considerations, the substitution of this residue severely reduces the stimulatory effect of ATP on the dehydratase activity of hSR, while maintaining the racemase activity almost unchanged. Methionine and alanine, due to their apolar side chains, likely interrupt the H-bond network that contributes to the bidirectional communication between the active site and the ATP-binding site. Indeed, our results demonstrate that Q89 is not only crucial for the “forward” allosteric control (i.e. ATP binding increases the catalytic efficiency of the dehydratase reaction and the affinity of active site ligands), but also for the “backward” control, in which occupation of the active site increases the affinity of ATP and abolishes cooperativity. The Q89A mutant, likely due to a local collapse of the protein structure brought about by the substitution of the bulky Met side chain with the methyl group of Ala, is less stable and more prone to precipitation. This hampered the in-depth characterization of this mutant, which, however, displays most of the functional features of Q89M-hSR. The Q89M mutation is associated with a higher intrinsic affinity for glycine (Table 2), but with a very small effect of glycine binding on the affinity for ATP. In hSR, the binding of glycine increases the apparent affinity of ATP by several folds and, most importantly, makes the binding non-cooperative. On the other hand, glycine has a more than 60-fold higher affinity for Q89M-hSR, but it brings about a mere 2.6-fold increase in the affinity for ATP when bound to the enzyme. Overall, our data suggest that plasticity in the structure of hSR is required to modulate the catalytic activity and specificity by ATP. Q89 seems to be important for the transitions between different states and its mutation to methionine stabilises a protein conformation with higher affinity for its ligands, but significantly smaller scope for conformational dynamics. Remarkably, we observed that, in the crystal structures of SR and its homologs solved to date, Q89 adopts different orientations depending on the protein/organism/ligation state. The superposition of the three-dimensional structures of *Sp*SR (bound to AMP-PCP or to serine), hSR (bound to malonate), and *St*TdcB (bound to CMP) demonstrates that all the five residues of the H-bond network overlap very well, with the exception of Q89 (Fig. S5). Indeed, this residue can adopt at least three distinct conformations, depending on the protein and its ligation state. The side chain of this residue in the structure of hSR in complex with malonate (pdb code: 3L6B) adopts two alternative conformations, with 50% occupancy each. This observation hints at a residue endowed with large conformational mobility, which might be required by its role as a “gate” in the path from the active site to the allosteric site and vice versa. It has been suggested that, upon simultaneous substrate and nucleotide binding, the conformational change might lead to a reorganization of the H-bond network between PLP and nucleotide site allowing Q89 (Q87 in *Sp*SR) to form a direct H-bond with the 2′ OH group of AMP-PCP^14^. Taken together, these data suggest that Q89 is highly dynamic and seems to have a crucial role in the allosteric communication.

**Figure 6:**
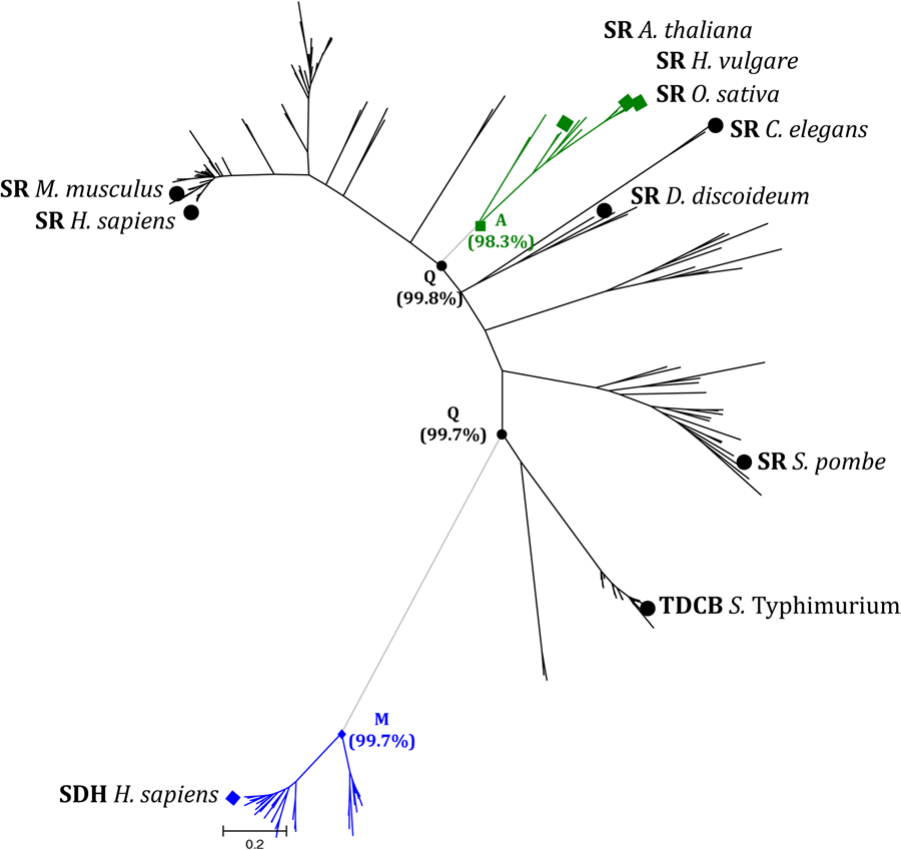
Ancestral state reconstruction of Q89 in the SR phylogeny. The unrooted maximum-likelihood tree of SR, SDH, and TdcB was used to infer the probability of ancestral states at position 89 of hSR as reported in parenthesis for selected nodes. Experimentally characterized proteins are indicated by symbols. Accession numbers are reported in Fig. S1.

The role played by Q89 stimulated the investigation of the evolutionary history of this position both in hSRs and structural/functional homologs (Fig. 6 and Fig. S1). The analysis was aimed at disclosing which amino acid was present at that position in a reconstructed ancestral sequence and which amino acid was the most probable at nodes, where descendants split from a common ancestor. A maximum-likelihood reconstruction indicated that the ancestral hSR sequence carries a glutamine at position 89, thus supporting the notion that regulation of hSR activity by ATP is ancient. SDH and hSR are evolutionary related and racemisation activity evolved as a side reaction of the dehydratase function^4^, however SDH activity is not modulated by ATP. Probably, ATP binding is a mandatory step in racemase evolution. Interestingly, this position underwent single, specific mutational events at the divergence of SDHs (99.8% probability to have a methionine at this position) and of plant SRs (98.1% probability to have an alanine at this position) that correspond to the loss of control of activity by nucleotides. No other mutational events were identified, and this further supports the notion that the position is strictly conserved depending on the function of the protein. The loss of activity control of plant hSR by ATP is particularly intriguing, since its functional relevance is unknown. The obvious question is whether ATP binding was lost together with the modulating function. This issue is not simply addressed by the conservation analysis of the residues of the ATP binding site. In fact, in hSR 14 residues contribute to the formation of the ATP-binding pocket^14^ and are conserved among yeast and mammalian sequences. In plant hSRs, some residues are different whereas some others, namely seven, are conserved (Fig. S6). Among mutated residues, A115 and R275 (*Sp*SR numbering) are substituted by N and T/M/I, respectively, in the 83% of analysed sequences. Since the adenine ring of ATP is sandwiched between A115 and R275 in *Sp*SR and a similar function is carried out by A116 and R276 in *St*TdcB, it might be possible that the mutation of these two residues is sufficient to decrease the affinity of the protein for the nucleotide at physiological concentrations. One might wonder what is the role of N84, E281 and N311, since they are conserved in all the sequences analysed, from both hSR and structural homologs. However, based on phylogenetic and functional considerations, they are supposed not to directly contribute to allosteric communication. Indeed, N84 and N311 are directly involved in PLP binding and are thus likely to play different roles in the protein. E281 (E283 in hSR) does not directly interact with ligands and is a good candidate to investigate the involvement of conserved residues in allostery. We introduced the E281A mutation successfully but were not able to express the protein in soluble form, an indirect experimental evidence that the residue might play a structural role and has been conserved even in those organisms where the allosteric control of activity by nucleotides was lost.

The physiological implications for the modulation of SR activity by ATP have been the object of pioneering studies^5^. It was suggested that D-Ser dehydration was likely a key activity for the control of D-Ser concentration in those brain areas that lack amino acid oxidase. Our results add new cues to the intriguing role of the dehydratase activity of this enzyme and Q89 mutants might indeed be useful to investigate, in cellular models, the role of ATP in D-Ser production and degradation. In addition, understanding how the modulation of SR activity by ATP affects D-Ser production could have an impact on our ability to identify more effective modulators of this potential drug target.

## Methods

### Materials

Reagents were purchased from Sigma-Aldrich and used as received. DpnI, NcoI were from New England Biolabs® Inc (Ipswich, Massachusetts, USA). Tris (2-carboxyethyl) phosphine (TCEP) was purchased from Apollo Scientific (Denton, Manchester, UK) and dithiothreitol (DTT) from Bio-Rad (Hercules, California, USA).

All experiments, if not otherwise indicated, were carried out in buffer A, composed of 50 mM triethanolamine (TEA), 150 mM NaCl, 2 mM MgCl_2_, 50 µM PLP, pH 8.0.

### Structure and sequence alignment

Structural alignments and pictures were made using Pymol 1.3^38^. The sequences searches were performed with BLAST^39,40^ against the UniProt^41,42^ and NCBI Refseq database using the sequence of human serine racemase (Q9GZT4) as query. Searches with Pattern Hit Initiated BLAST (PHI-BLAST)^43^ and the pattern S-X-G-N-X-X-{Q} were carried out in order to find all possible hSRs with an amino acid different from glutamine at position 89. hSR, SDH, and TdcB sequences were selected on the basis of sequence identity and evolutionary distance with experimentally validated proteins. Sequences were assessed as belonging to fold-type II by the Analyse function within the B6 Database^44^. Sequence alignments were carried out using Clustal Omega^45^ with default parameters. Similarity scores were calculated by the ESPript program^46^ using the Blosum62 matrix set at global score of 0.25. Sequence logos for positions associated with the hydrogen bond network were generated with WebLogo3^47,48^ using the corresponding columns extracted from the multiple sequence alignment. Residue conservation was expressed as reduction of uncertainty with respect to the maximum entropy per site (log_2_ 20 ≈ 4.32 bits) for protein sequences^47,48^. The phylogenetic tree was constructed using the maximum-likelihood method and the JTT (Jones-Taylor-Thornton) evolutionary model implemented in MEGA 7^49^. Posterior probabilities at each site for ancestral nodes were calculated using codeml program in the Phylogenetic Analysis by Maximum Likelihood (PAML) package^50^, based on the sequence alignment and phylogenetic tree of SR, SDH, and TdcB proteins.

### Site-directed mutagenesis

Mutagenesis was performed on the hSR gene cloned in pET28a^51^. Q89M-hSR was obtained with the QuikChange kit (Agilent, Santa Clara, CA, USA) using primers that, in addition to the mutation for the substitution of the desired amino acid, also carry a substitution to remove a restriction site and thus facilitate the screening of recombinants. The primers, purchased from Eurofins Genomics (Germany), have the following sequences:

5′-GCAGTGGAAACCACGGCA**TG**GCTCTCACCTATGCTG-3′

5′-CAGCATAGGTGAGAGC**CA**TGCCGTGGTTTCCACTGC-3′

where the underlined nucleotides indicate the mutation that removes the restriction site for NcoI and the nucleotides in bold indicate the mutations that introduce the Gln to Met substitution.

The correct incorporation of the mutation was assessed by DNA sequencing of the gene.

Q89A-hSR was obtained, with minor modifications, following the method developed by Liu and Naismith^52^, that exploits primers containing non-overlapping sequences at their 3′ ends.

The primers, purchased from Eurofins Genomics (Germany), have the following sequences:

5′-*ATGGC****GC****G*GCTCTCACCTAT-3′

5′-*C****GC****GCCAT*GGTTTCCACTGC-3′

where the nucleotides in bold indicate the mutations that introduce the Gln to Ala substitution and the primer-primer overlapping sequences are in italic.

PCR reaction was carried out with Phusion High-Fidelity DNA polymerase (Thermo Scientific, Waltham, MA, USA), in accordance with manufacturer’s instructions.

The correct incorporation of the mutation was assessed by DNA sequencing of the gene.

### Protein expression and purification

Q89M-hSR and Q89A-hSR were expressed as a hexa-His tagged fusion protein encoded from the pET28a-derived plasmid transformed into *E. coli* BL21 CodonPlus (DE3)-RIL cells (Agilent Technologies, Santa Clara, CA, USA), previously transformed with plasmids encoding GroEL and GroES chaperonins. These chaperonins were selected from the Chaperone Plasmid set, Takara^®^, as they increased the expression of soluble hSR. Cells were grown at 37 °C in Luria-Bertani medium supplemented with 50 µg/ml kanamycin and 20 µg/ml chloramphenicol. When the cells reached an OD at 600 nm of ∼0.5, 0.5 mg/ml arabinose and 1 ml/l benzyl alcohol were added to the medium and the growth temperature was lowered to 20 °C. After 20 minutes isopropyl β-D-1-thiogalactopyranoside (IPTG) was added to a final concentration of 0.05 mM and the culture was further incubated at 20 °C for about 20 hours. Cells were harvested by centrifugation and the pellet was resuspended in lysis buffer containing 50 mM Na_2_HPO_4_, 150 mM NaCl, 5 mM TCEP, 50 µM PLP, 0.2 mM phenylmethylsulfonyl fluoride (PMSF), 0.2 mM benzamidine, 1.5 µM pepstatine at pH 8.0. Cells were disrupted by treatment with lysozyme followed by sonication. The protein was purified from the crude extract by IMAC on TALON® resin (Clontech, Mountain View, CA, USA). The final protein solution, with a concentration of about 5 mg/ml, was flash-frozen in liquid nitrogen and stored at −80 °C. Protein purity was assessed by densitometry of Coomassie blue-stained bands of a SDS-PAGE gel, using a ChemiDoc Image System^TM^ (Bio-Rad, Hercules, CA, USA).

### Spectroscopic measurements

Absorbance spectra were collected using a Varian Cary4000 spectrophotometer (Agilent Technologies, Santa Clara, CA, USA) on solutions containing 34 μM hSR, 50 mM TEA pH 8.0, at 20.0 ± 0.5 °C. Fluorescence emission spectra upon excitation at 412 nm were collected using a FluoroMax-3 fluorometer (HORIBA Jobin Yvon, Kyoto, Japan) at 20.0 ± 0.5 °C. The solution for fluorescence measurements contained 2.7 µM hSR in buffer A with 5 mM TCEP. Binding of either glycine or ATP was measured by direct titrations of the protein solution in the range 0–1.2 mM and 0–1.0 mM, respectively. Added volume did not exceed 20% of the initial volume and the spectra were corrected for dilution. Spectra were corrected for buffer contribution. Far-UV circular dichroism spectra (195–260 nm) were collected using a JASCO J-715 spectropolarimeter (Easton, MD, USA). Measurements were performed at 20.0 ± 0.5 °C on a solution containing 4 μM hSR, 20 mM NaH_2_PO_4_ pH 7.5. Each spectrum is the average of three independent measurements.

### Dehydratase activity assays

The initial velocity of L-serine dehydration by hSR was monitored by a coupled assay with lactate dehydrogenase (LDH)^5^ as previously reported^53^. Nicotinamide adenine dinucleotide (NADH) consumption was followed at 340 nm using a Varian Cary4000 spectrophotometer. The assay solution in buffer A contained 5 mM DTT, 30 U/ml LDH and 300 μM NADH. The concentration of magnesium used in the assay was always higher than or equal to the concentration of ATP. If not otherwise stated, it was set to 2 mM. The protein concentration used in the assay for L-Ser dehydration was 0.4 and 1.6 μM in the presence and absence of 2 mM ATP, respectively. The concentration used in the assay for D-Ser dehydration was 3.1 and 6.0 μM in the presence and absence of ATP, respectively.

### Racemase activity assay

The formation of D-serine as a function of L-serine concentration was determined using a discontinuous assay, based on oxidation of D-serine by the enzyme DAAO (Sigma Aldrich, A5222)^18,37,54^. This reaction produces hydrogen peroxide, that is used by the horseradish peroxidase to oxidize *o*-dianisidine dihydrochloride to form a chromophoric product. After the addition of sulphuric acid to increase the solubility, the concentration of this product can be measured at 550 nm. L-serine at various final concentrations was added to buffer A containing 1.5 µM hSR, in the absence and presence of 2 mM ATP. The reaction mixture was incubated at 37 °C and aliquots were periodically removed to determine D-serine concentration. L-serine was purified from any D-serine contaminations through 48 hours of incubation with 400 U/ml of DAAO and 100 U/ml of catalase, which were subsequently thermally inactivated^18^. Absolute D-serine concentrations were determined using calibrations curves obtained by reacting known D-serine concentrations with DAAO. Absorbance signal was acquired by a Halo LED 96 plate reader (Dynamica Scientific, UK).

### Data analysis

The half-maximal activation (EC_50_) caused by ATP binding to hSR was calculated with Eq. (1):

1 $$\frac{{v}_{i}}{{v}_{0}}=a\cdot \frac{[ATP]}{E{C}_{50}+[ATP]}$$

where *v*_*i*_ is the velocity of the reaction catalysed by the enzyme in presence of a defined ATP concentration, *v*_0_ is the velocity of the reaction catalysed by the enzyme in the absence of ATP and *a* is the difference between the maximum and the basal activity. The dissociation constant (K_D_) of ligands for hSR calculated by fluorimetric titrations was calculated with Eq. (2):

2 $$y={y}_{0}+a\cdot \frac{[L]}{{K}_{D}+[L]}$$

where *y* is the fluorescence emission intensity, *[L]* is the ligand concentration, *y*_0_ is an offset and *a* is the amplitude. The fluorescence emission intensity was collected at 480 nm for glycine titration, at 492 nm for ATP titration and at 478 nm for ATP titration in the presence of glycine. The kinetic parameters were calculated using the Michaelis –Menten equation:

3 $${v}_{0}=\frac{{V}_{max}\cdot [S]}{{K}_{m}+[S]}$$

where v_0_ is the initial enzymatic rate, V_max_ is the rate at substrate saturation, K_m_ is the Michaelis constant and [S] is the substrate concentration. Catalytic efficiency for the dehydratase activity of Q89A-hSR on D-Ser was calculated at [D-Ser] < K_m_, where equation (3) becomes ${v}_{0}\cong \frac{{V}_{max}}{{K}_{m}}$.

### Data availability statements

All data generated or analysed during this study are included in this published article (and its Supplementary Information files).

## Electronic supplementary material

Supplementary Information
