# Supplementary material for: Glutamine 89 is a key residue in the allosteric modulation of human serine racemase activity by ATP
Source: Sci Rep. 2018 Jun 13;8:9016. doi: 10.1038/s41598-018-27227-1 (PMC5998037; doi:10.1038/s41598-018-27227-1)
Supplement: Supplementary file 1 — Supplementary Information [file 41598_2018_27227_MOESM1_ESM.pdf]

SUPPLEMENTARY INFORMATION

Glutamine 89 is a key residue in the allosteric modulation of human serine racemase activity by ATP

Andrea V. Canosa[1,], Serena Faggiano[1,2,], Marialaura Marchetti[3], Stefano Armao[3], Stefano Bettati[4], Stefano Bruno[1], Riccardo Percudani[5], Barbara Campanini[1*], Andrea Mozzarelli[1,2]

[1]Dipartimento di Scienze degli Alimenti e del Farmaco, Università di Parma, Parma, Italy

[2]Istituto di Biofisica, Consiglio Nazionale delle Ricerche, Pisa, Italy

[3]Centro Interdipartimentale Biopharmanet-tec, Università degli Studi di Parma, Parma, Italy

[4]Dipartimento di Medicina e Chirurgia, Università di Parma, Parma, Italy

[5]Dipartimento di Scienze Chimiche, della Vita e della Sostenibilità Ambientale, Università di Parma, Parma, Italy

[*]barbara.campanini@unipr.it

[+]these authors contributed equally to this work

Supplementary Figure S1.

Maximum-likelihood tree of SR, SDH, and TdcB.

The phylogenetic tree has been rooted using SDH as outgroup. Sequences are indicated by species names and Uniprot or GenBank accession numbers. Branches are colored according to the reconstructed state at position 89 in hSR as specified in Figure 6.

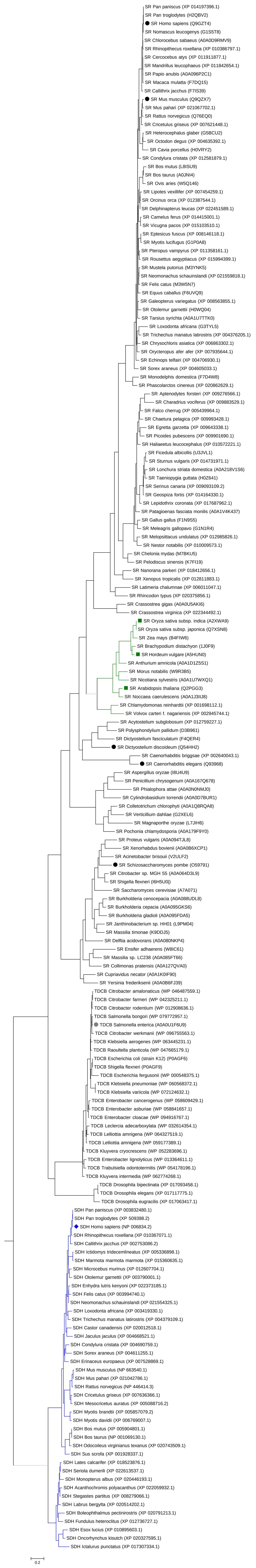

Supplementary Figure S2

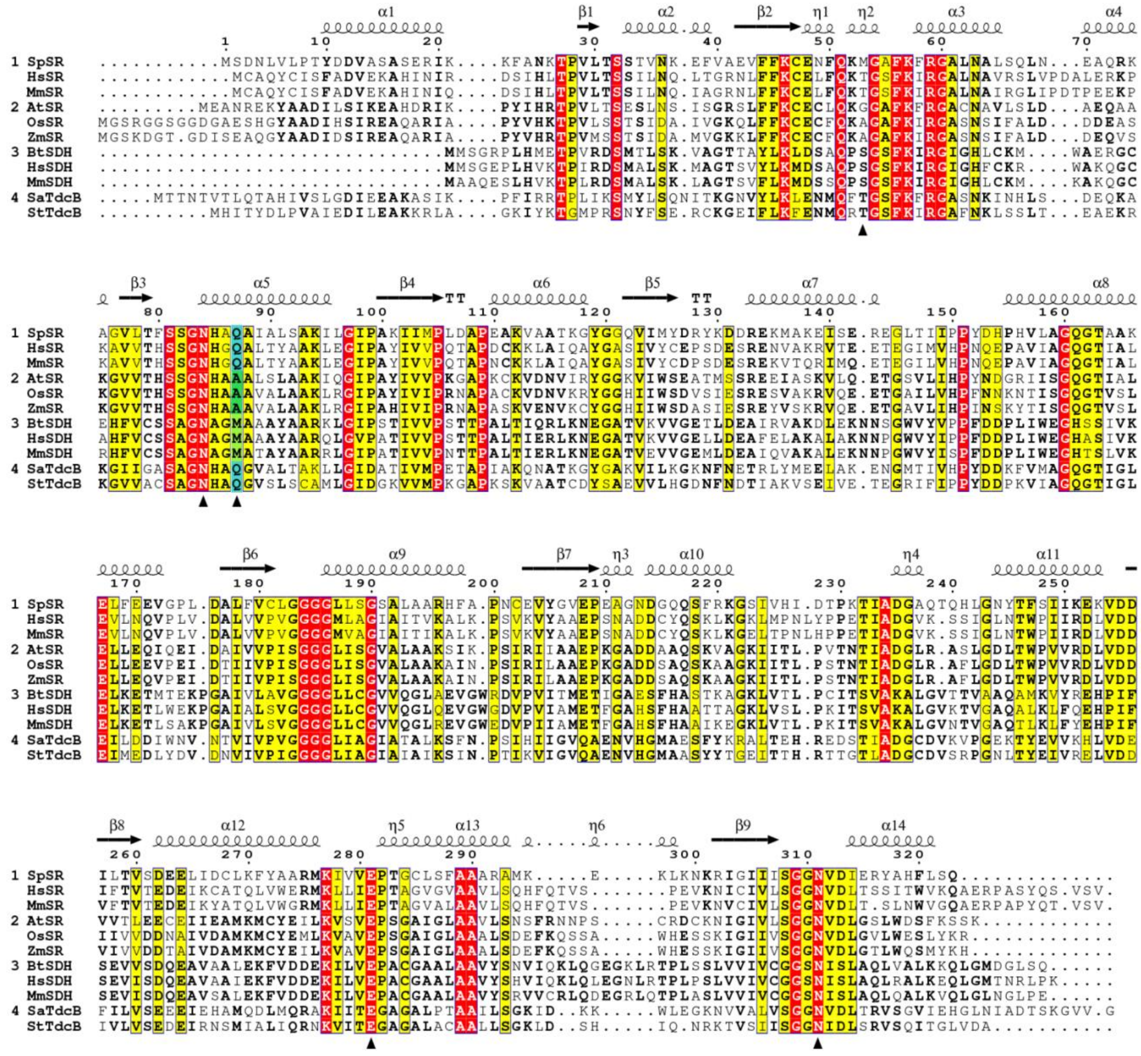

Amino acid sequence alignment of hSR with structural and functional homologs. The sequences of SR from fission yeast and mammals (group 1), SR from plants (group 2), SDH from mammals (group 3) and TdcB from bacteria (group 4) were aligned using Clustal Omega with Blosum matrix set at default parameters. Similarity scores were calculated by the ESPript program[1] using the Blosum62 matrix set at global score of 0.25. Identical residues are highlighted in red boxes; similar residues are boxed in yellow; similar residues within a group are in black bold characters. Residues involved in the H-bond wire that connects the active site with the ATP binding site[2] are indicated by dark triangles below the alignment. Residues at position 87 (*Sp*SR numbering) are shaded in green according to the group division. Secondary structure elements depicted above the alignment are derived from the crystal structure of *Sp*SR (pdb: 1WTC). *Sp*SR: *Schizosaccharomyces pombe* SR; *Hs*SR: *Homo sapiens* SR; *Mm*SR: *Mus musculus* SR; *At*SR: *Arabidopsis thaliana* SR; *Os*SR: *Oryza sativa* SR; *Zm*SR: *Zea mays* SR; *Bt*SDH: *Bos taurus* SDH; *Hs*SDH: *Homo sapiens* SDH; *Mm*SDH: *Mus musculus* SDH; *Sa*TdcB: *Staphilococcus aureus* TdcB; *St*TdcB: *Salmonella enterica* serovar Typhimurium TdcB.

Supplementary Figure S3

A

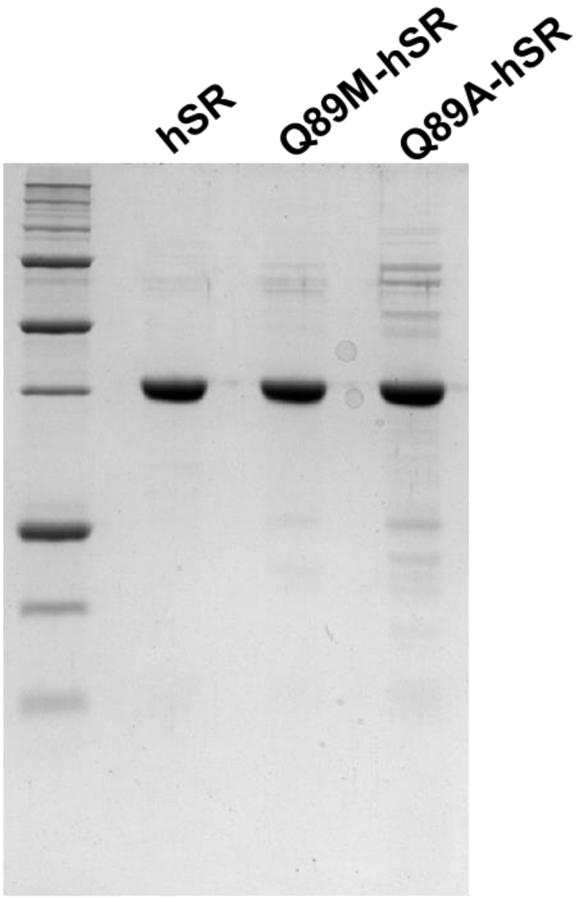

B

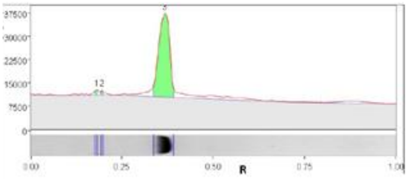

Band No.	Band Label	Mol. Wt. (KDa)	Relative Front	Adj. Volume (Int)	Volume (Int)	Abs. Quant. (ng)	Rel. Quant.	Band %
3		37.0	0.368	54,581,495	88,480,395	534.0	N/A	97.8
2		64.2	0.196	467,680	4,580,420	242.2	N/A	0.8
1		68.2	0.180	736,833	5,869,068	243.7	N/A	1.3

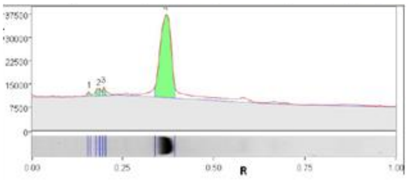

Band No.	Band Label	Mol. Wt. (KDa)	Relative Front	Adj. Volume (Int)	Volume (Int)	Abs. Quant. (ng)	Rel. Quant.	Band %
4		36.9	0.369	54,445,615	89,065,153	533.3	N/A	94.2
3		63.5	0.199	1,161,616	7,461,076	245.9	N/A	2.0
2		67.0	0.185	1,637,591	9,970,511	248.5	N/A	2.8
1		74.1	0.158	574,409	6,756,317	242.8	N/A	1.0

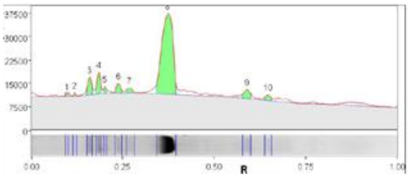

Band No.	Band Label	Mol. Wt. (KDa)	Relative Front	Adj. Volume (Int)	Volume (Int)	Abs. Quant. (ng)	Rel. Quant.	Band %
10		18.1	0.648	1,786,585	13,374,858	249.3	N/A	2.4
9		22.2	0.591	3,297,934	17,579,080	257.5	N/A	4.5
8		36.6	0.374	54,442,850	91,353,072	533.3	N/A	73.7
7		49.1	0.269	1,479,433	17,261,342	247.7	N/A	2.0
6		54.7	0.239	2,482,891	17,123,487	253.1	N/A	3.4
5		62.7	0.202	958,428	7,601,064	244.8	N/A	1.3
4		66.6	0.186	4,368,384	14,178,604	263.2	N/A	5.9
3		73.2	0.161	4,104,366	14,774,580	261.8	N/A	5.6
2		89.0	0.119	372,406	8,639,914	241.7	N/A	0.5
1		98.5	0.099	605,851	6,763,743	242.9	N/A	0.8

SDS-PAGE and densitometric analysis of hSR, Q89M-hSR and Q89A-hSR. (A) SDS-PAGE of hSR, Q89M-hSR and Q89A-hSR preparations used in this work. The molecular weight marker is Precision Plus Protein™ Prestained Standard (BioRad). (B) Densitometric analysis was carried out using Image Lab™ software (BioRad) and indicated that hSR is more than 98% pure, Q89M-hSR is more than 94% pure and Q89A-hSR is about 74% pure.

Supplementary Figure S4

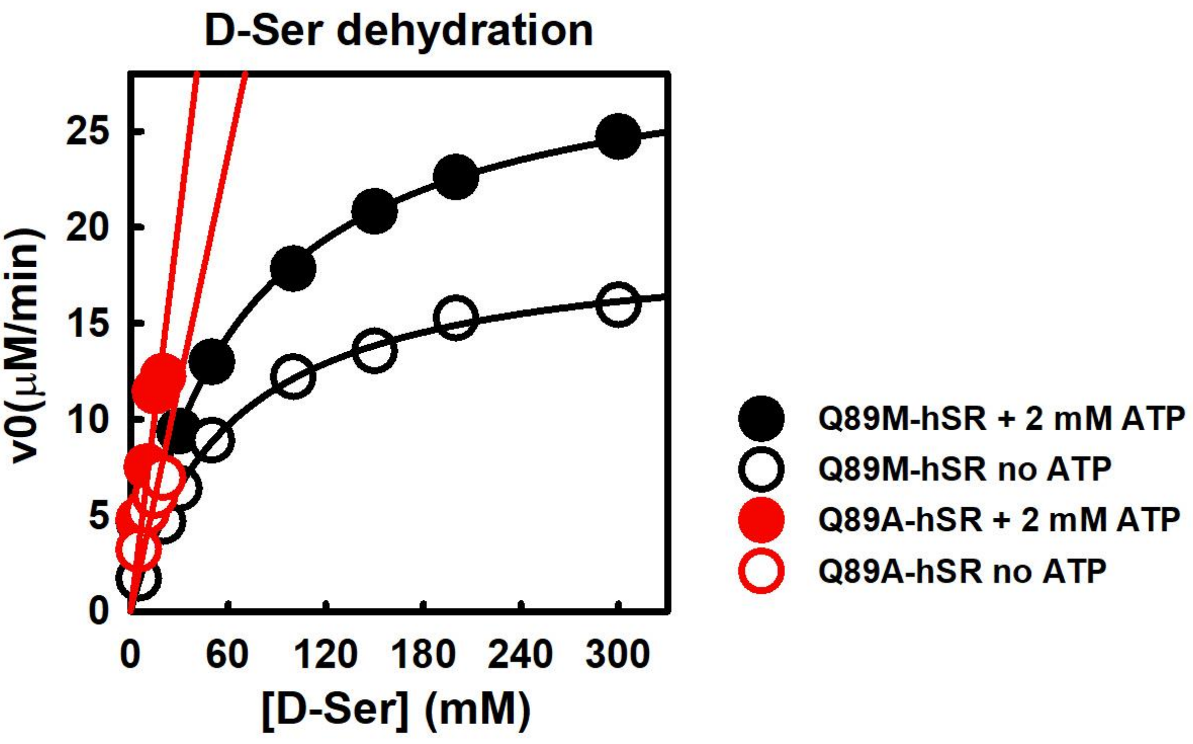

Dehydratase activity on D-Ser of Q89M-hSR and Q89A-hSR. The dependences of the initial rates on D-Ser concentration were fitted to equation (3) for Q89M-hSR and to a straight line with slope= V_{max}/K_m for Q89A-hSR. Dependences were collected either in the presence or absence of 2 mM ATP. Calculated kinetic parameters are reported in Table 1. Error bars are not visible since they are smaller than the data points.

Supplementary Figure S5

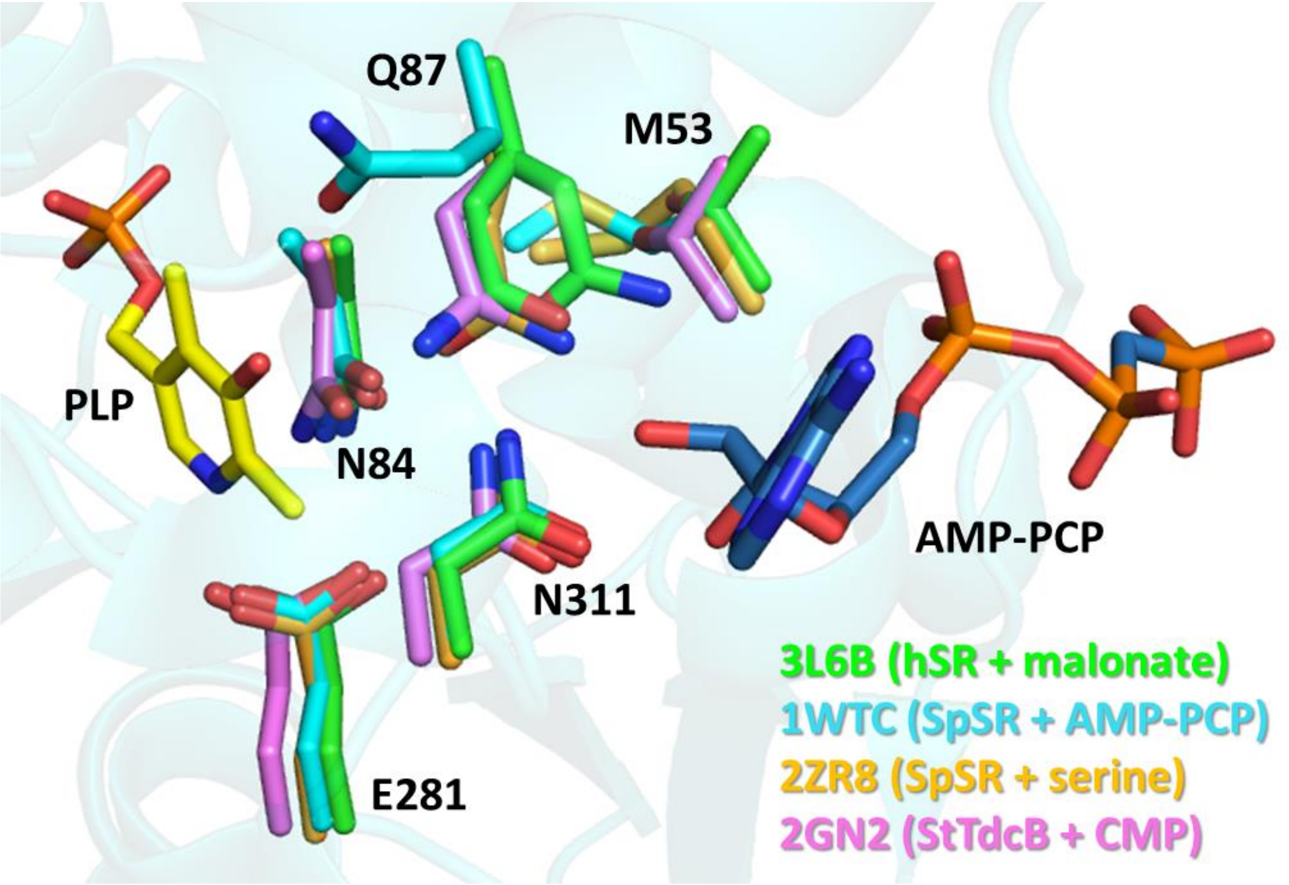

Conformations of the five amino acids on the H-bond network in different structures. The structures of hSR in complex with malonate, *Sp*SR in complex with either AMP-PCP or serine and StTdcB in complex with CMP were overlaid using Pymol and the residues of the H-bond network represented in stick-mode. Numbering is based on *Sp*SR sequence (*Sp*SR Q87 corresponds to hSR Q89).

Supplementary Figure S6

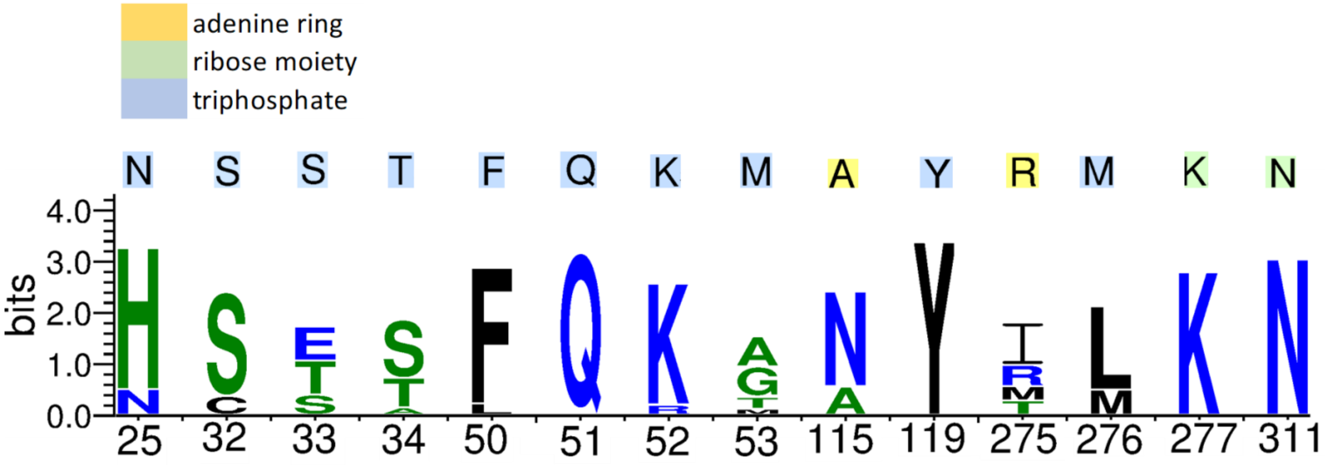

Conservation analysis of ATP-binding site residues in plant SRs. Conservation analysis of the residues forming the ATP-binding site of SpSR (colored yellow, green and cyan depending on the part of the ATP they interact with) in plant SRs (logo plot).

REFERENCES

1 Gouet, P., Courcelle, E., Stuart, D. I. & Metoz, F. ESPript: analysis of multiple sequence alignments in PostScript. *Bioinformatics* **15**, 305-308, doi:10.1093/bioinformatics/15.4.305 (1999).

2 Goto, M. *et al.* Crystal Structure of a Homolog of Mammalian Serine Racemase from Schizosaccharomyces pombe. *J. Biol. Chem.* **284**, 25944-25952, doi:10.1074/jbc.M109.010470 (2009).
